# Technical Support by Smart Glasses During a Mass Casualty Incident: A Randomized Controlled Simulation Trial on Technically Assisted Triage and Telemedical App Use in Disaster Medicine

**DOI:** 10.2196/11939

**Published:** 2019-01-03

**Authors:** Andreas Follmann, Marian Ohligs, Nadine Hochhausen, Stefan K Beckers, Rolf Rossaint, Michael Czaplik

**Affiliations:** 1 Medical Technology Section Department of Anaesthesiology University Hospital RWTH Aachen Aachen Germany; 2 Docs in Clouds GmbH Aachen Germany; 3 Medical Direction Emergency Medical Service City of Aachen Aachen Germany

**Keywords:** augmented reality, disaster medicine, emergency medical service physician, mass casualty incident, Smart Glasses, telemedicine, triage

## Abstract

**Background:**

To treat many patients despite lacking personnel resources, triage is important in disaster medicine. Various triage algorithms help but often are used incorrectly or not at all. One potential problem-solving approach is to support triage with Smart Glasses.

**Objective:**

In this study, augmented reality was used to display a triage algorithm and telemedicine assistance was enabled to compare the duration and quality of triage with a conventional one.

**Methods:**

A specific Android app was designed for use with Smart Glasses, which added information in terms of augmented reality with two different methods—through the display of a triage algorithm in data glasses and a telemedical connection to a senior emergency physician realized by the integrated camera. A scenario was created (ie, randomized simulation study) in which 31 paramedics carried out a triage of 12 patients in 3 groups as follows: without technical support (control group), with a triage algorithm display, and with telemedical contact.

**Results:**

A total of 362 assessments were performed. The accuracy in the control group was only 58%, but the assessments were quicker (on average 16.6 seconds). In contrast, an accuracy of 92% (*P*=.04) was achieved when using technical support by displaying the triage algorithm. This triaging took an average of 37.0 seconds. The triage group wearing data glasses and being telemedically connected achieved 90% accuracy (*P*=.01) in 35.0 seconds.

**Conclusions:**

Triage with data glasses required markedly more time. While only a tally was recorded in the control group, Smart Glasses led to digital capture of the triage results, which have many tactical advantages. We expect a high potential in the application of Smart Glasses in disaster scenarios when using telemedicine and augmented reality features to improve the quality of triage.

## Introduction

Terrorist attacks, natural disasters, and major traffic accidents present challenges to emergency physicians around the world. The lack of information about those affected and injured and an initial difference between available and necessary resources require a quick overview of the overall situation. In disaster medicine, various strategies exist for assisting the management of mass casualties and prioritizing injured persons according to the need and available resources. Serious injuries require urgent treatment; immediate life-saving measures may be required. Slightly injured persons have to be cared for, but their transport from the damage area can be postponed owing to limited resources; this results in the need of prioritization of treatment and transport to a hospital—the procedure of triage [1]. The triage category is encoded in color and recorded with an individual identification number on a so-called “injured attachment card.”

In many countries around the world, different triage algorithms are established to help rescue service personnel and emergency physicians in assigning casualties to one of the triage categories [2]. Algorithms are different for doctors and nonmedical staff who perform the so-called “pretriage”; this is important for many reasons, including providing an overview of the reclaiming of additional personnel. The Primary Ranking for Initial Orientation in Rescue service (PRIOR) algorithm is a pretriage algorithm often used in Germany [3]. Qualitatively, patient consciousness, breathing, and circulation are addressed, which results in the case of nonpathological evaluation in further questions for individual triage; this can be divided into 3 categories as follows: severely injured with immediate treatment priority (category red or I); severely injured with appropriate transport priority (category yellow or II); and easily injured or uninjured (category green or III). However, various triage algorithms are often used incorrectly or not at all [4-6]; this results in incorrect assignments of triage categories and wrong prioritization. Subsequently, scarce resources cannot be used correctly, appropriate treatment priorities are neglected, and treatments delayed [7,8]. All of these are in contradiction with the principles of triage in disaster medicine [9]. From this, it can be concluded that technical support for triage is urgently needed. Therefore, we used the augmented reality and the potential of data glasses for this important task.

To provide technical support for this important phase in the case of mass casualty incidents (MCI), we have developed a triage app running on Smart Glasses (Recon Jet, Recon Instruments, Vancouver, BC, Canada) within the framework of the project Augmented Disaster Medicine (AUDIME), which was financed by the German Federal Government. The AUDIME project aims to develop new high-tech strategies, including apps for Smart Glasses, for the technical support of an MCI [10]. Data are displayed on a small monitor on the Smart Glasses, the video stream of an integrated camera is used for telemedical support. With simple operating gestures provided on an optical touchpad on the Smart Glasses, menu items can be selected, and simple manual entries can be made.

This study aims to evaluate the feasibility of various technical methods for triage support using Smart Glasses. The average duration of a screening process and the accuracy of the assignment to one of the listed triage categories are the primary target parameters.

## Methods

### Android App

**Figure 1:**
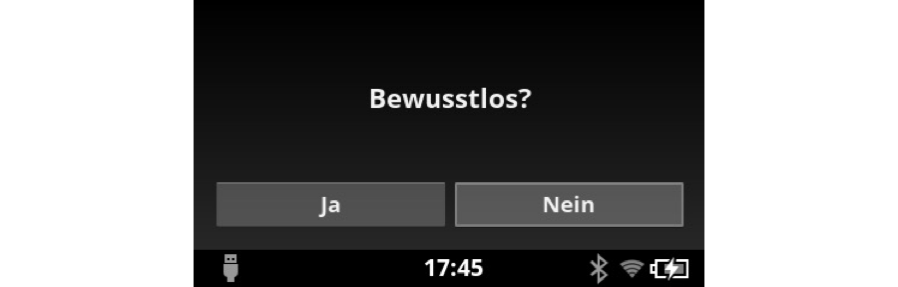
Question regarding unconsciousness is asked (“yes” or “no”) on the Smart Glasses screen.

For Smart Glasses, an app was developed together with our project partner (Tech2go GmbH, Hamburg, Germany) for use on Android devices. Using several menu levels, information can be displayed to task forces, and appropriate support can be offered. Triage support was achieved through the display of PRIOR as an example of one of the various triage algorithms (Figure 1). With simple hand movements above the optical touchpad, the decision tree can be processed, and the result of the triage is displayed. The result is recorded digitally and assigned to the individual ID of the patient appendix card through a photo.

### Telemedical Support

**Figure 2:**
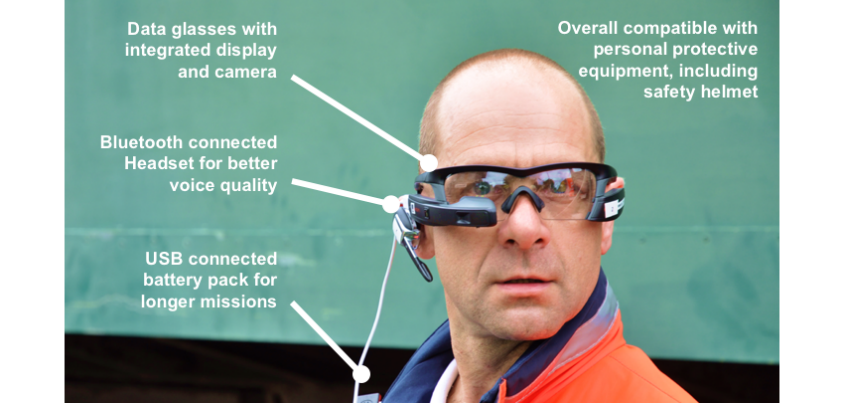
The subcomponents of the triage technical support system.

Another method for investigating technical support provided through Smart Glasses is telemedicine. In Aachen, Germany, this has been used for many years in individual medical emergency care in the routine prehospital rescue service [11-15]. A telemedical-connected emergency medical service (EMS) physician can offer medical assistance to the ambulance staff for making difficult decisions. Telemedicine has not yet been used in disaster medicine. Therefore, the camera of the Smart Glasses was used in this project to provide information about the MCI site through a live video transmission to a tele-EMS-physician. The physician was thus able to gain an impression of the situation and carry out triage collaboratively with the on-site team and assign each patient to a triage category. For this purpose, a separate tele-senior-EMS-physician software (Tele-LNA, Docs in Clouds GmbH, Aachen, Germany) was developed. Again, a digital recording of the triage results was retained. The audio connection was achieved through Voice over Internet Protocol. For better voice quality, a Bluetooth-connected headset was used. Optionally, a battery pack can be connected to optimize the battery capacity of the Smart Glasses even for longer periods of use (Figure 2).

The function of the AUDIME system requires a local Wi-Fi network, which can be spanned by local access points on site; although this technology is still unusual at a deployment site, we assume that many rescue vehicles in the future will have such technology installed to ensure interoperability between rescue and medical technology. Alternative concepts, such as the use of the mobile network, are also conceivable. Using a secure data connection, all collected data are transferred to an information integration layer of the AUDIME server, analyzed, and made available for use on Smart Glasses or other devices, such as tablet personal computers.

### Study Design

For evaluation in a randomized triage study, a simulation was set up on the grounds of the Fire Brigade and Rescue Training Center in Frankfurt with the approval of the Local Ethics Committee (Aachen, Germany, EK 185/17), which represented an explosion in a row of residential buildings. A total of 12 professional actors mimed patients with different injuries (Table 1). Two of each injury were of identical make-up. The actors were required to exaggerate the case once and then understate it once.

A total of 31 paramedics were available as subjects for the simulation study. Each had sufficient experience of at least 2 years being in practice in EMS. The inclusion criteria for participation, in addition to requisite vocational training, was sufficient eyesight with contact lenses or without visual aids, as the Smart Glasses do not accommodate the wearing of prescription or reading glasses; therefore, this was considered an exclusion criterion. Participants were randomly assigned to 1 of the 3 groups as follows: group 1 was asked to perform an individual triage without further aids and document the results in a tally (control group); group 2 was asked to use the PRIOR algorithm provided by the Smart Glasses app; and group 3 was asked to contact a tele-senior EMS physician to collaboratively carry out the triage of an injured through a video streaming through the integrated camera of the data glasses. Group assignments were unknown to subjects in advance (blinding). The tele-EMS physician had specialist training in anesthesia, intensive care, and emergency medicine, as well as many years of experience as a regular EMS physician.

To ensure the same level of knowledge regarding triage algorithms, all paramedics, including the control group, were trained on the PRIOR algorithm half an hour prior to the simulation (no one knew it before). In addition, all paramedics were provided with the PRIOR algorithm as a pocket card. Subsequently, they were assigned to the method they should use for the triage depending on the study group to which they belonged. The size of each group was based on the estimated time spent on the experiences of the first pilot study.

**Table 1:** The overview of injury patterns of simulation patients.

Number	Injury	Type of simulation	Triage category
1	Unconsciousness	N/A^a^	I/red
2	Unconsciousness	N/A	I/red
3	Open lower leg fracture	Understated	II/yellow
4	Open lower leg fracture	Exaggerated	II/yellow
5	Piling lower leg	Understated	II/yellow
6	Piling lower leg	Exaggerated	II/yellow
7	Deep thigh wound, no bleeding	Understated	II/yellow
8	Deep thigh wound, no bleeding	Exaggerated	II/yellow
9	Cuts face	Understated	III/green
10	Cuts face	Exaggerated	III/green
11	Head wound	Understated	III/green
12	Head wound	Exaggerated	III/green

^a^Not applicable.

Successively, subjects completed the screening process according to their group assignment. In each case, they were unobtrusively accompanied by an auxiliary who documented the duration of triage and the selected category. Then, the primary target parameters were assessed—duration and accuracy of triage. If the selected triage category did not agree with the correct category, so-called “major deviations” were defined for selected green (III) instead of red (I) or red (I) instead of green (III) category. Subsequently, paramedics of the 2 technical supported groups completed a usability questionnaire. In addition, the feelings of safety of all participating teams and their individual opinions on the executed triage processes are inquired. The data from the subject survey were defined as further outcome parameters.

### Statistical Analysis

Data analysis was performed on the nonparametric distribution of primary outcome parameters using the Mann-Whitney U test for independent samples (significance level, *P*=.05). We used SPSS Statistics software, version 23 (IBM) for statistical evaluation and data backbone. All data are stated as median, interquartile range (IQR), or minima to maxima, respectively. The evaluation of the secondary outcome parameters was descriptive.

## Results

### User Results

In total, 362 individual triages were performed by 31 paramedics (Table 2); 20 paramedics conventionally triaged 240 patients with manual coverage of the triage category (control group), 7 paramedics triaged a total of 84 patients with an indication of the PRIOR algorithm in the Smart Glasses, and 4 paramedics performed a total of 38 triages along with a tele-senior EMS physician.

### Duration of Triage

**Figure 3:**
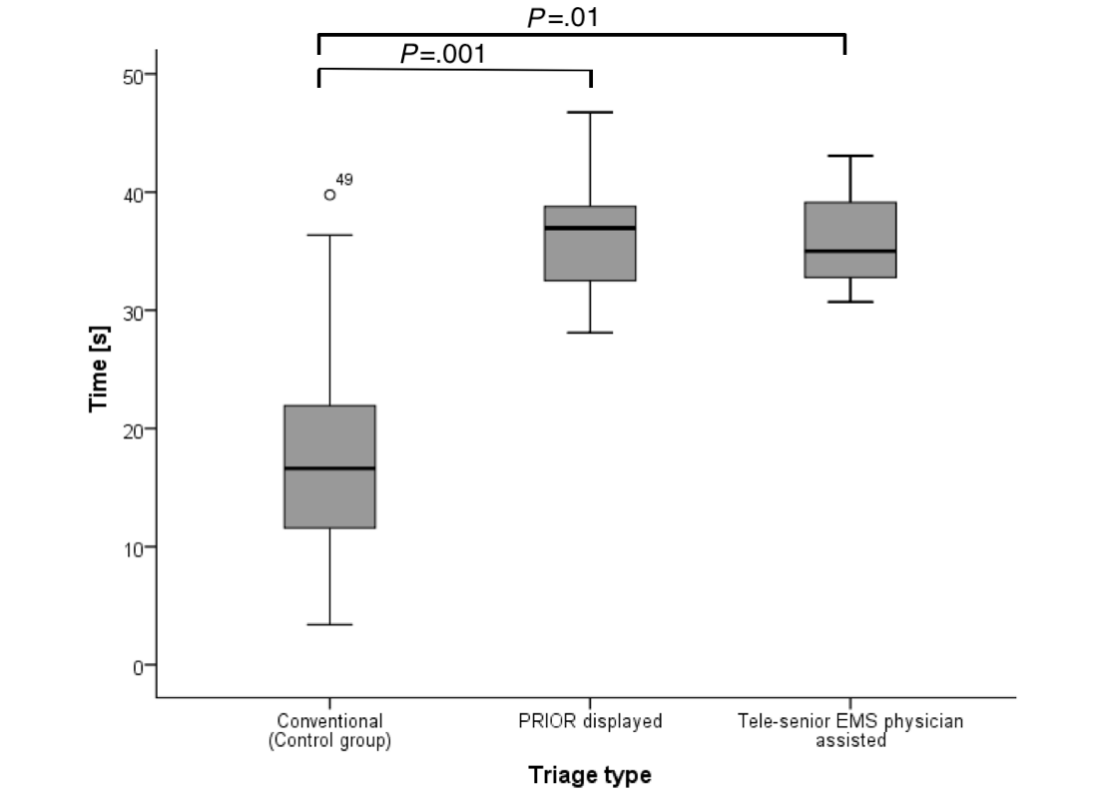
Time (CI) required per triage type. Circles with numbers denote outliers. PRIOR: Primary Ranking for Initial Orientation in Rescue service; EMS: emergency medical service.

**Figure 4:**
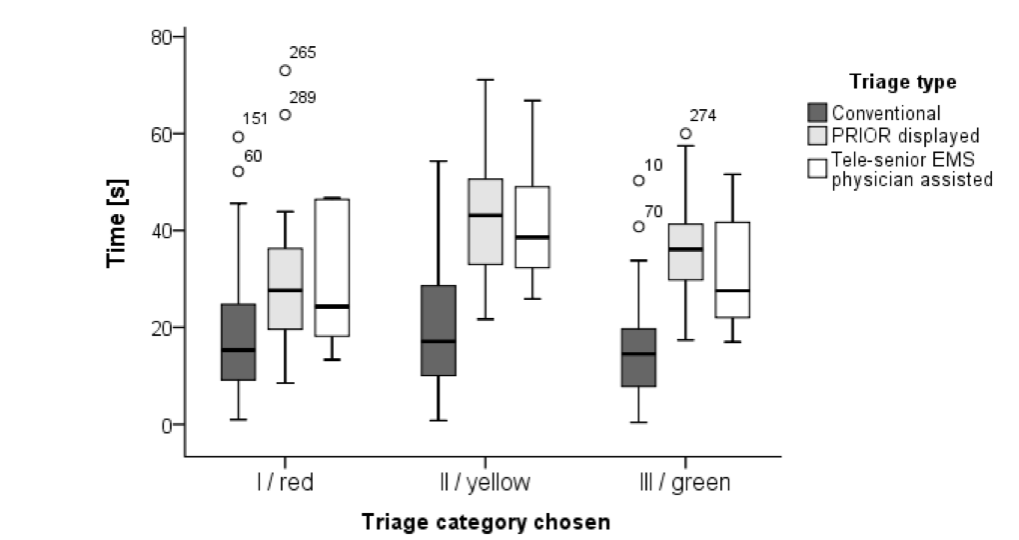
Time (CI) required for a triage in each individual category (from I, seriously injured to III, slightly injured) per each triage group Circles with numbers denote outliers. PRIOR: Primary Ranking for Initial Orientation in Rescue service; EMS: emergency medical service.

While the average triage time in the control group (conventional triage with manual coverage of the triage category in a tally) was 16.6 seconds (IQR 11.3-23.6), screening with technical support was longer. The average triage time when using the PRIOR in the Smart Glasses was 37.0 seconds (IQR 28.7-40.4; *P*=.001), a triage along with the tele-senior EMS physician averaged 35.0 seconds (IQR 31.7-41.1; *P*=.01). Thus, a triage with technical support and digital capture took markedly longer than conventional triage (Figure 3). The figure includes time required per triage type: Conventional triage, Triage with the PRIOR display in data glasses, and Triage with tele-senior EMS physician by the integrated camera of data glasses. Only technically supported procedures involve a digital capture of the category. Looking at the individual triage categories, the increased time requirements of the technically supported groups, especially in category II and III, were observed (Figure 4).

### Quality of Triage

**Figure 5:**
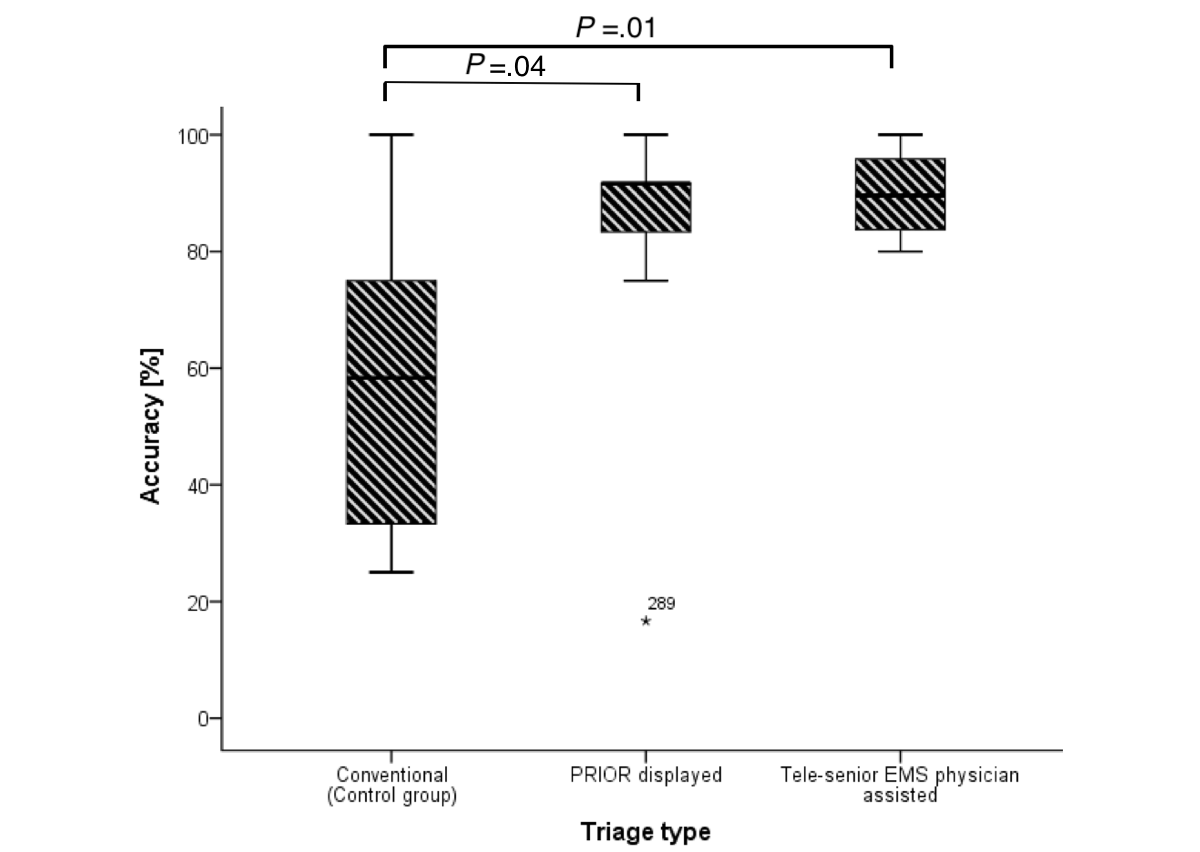
Accuracy (CI) of the chosen triage results per triage group. Numbers denote outliers. PRIOR: Primary Ranking for Initial Orientation in Rescue service; EMS: emergency medical service.

The correct assignment to a triage category was the primary outcome parameter. In the conventional triage, subjects reached an average accuracy of 58% (IQR 33%-75%) of the triage results. The quality of the triages could, however, be markedly increased with technical support. The accuracy was significantly increased to 92% (IQR 75%-92%) for the triage with the PRIOR Smart Glasses app (*P*=.04) and to 90% (IQR 82%-98%; *P*=.01) with tele-senior-EMS-physician assistance by data glasses (Figure 5). Deviations of 2 category ranks were found only in the control group. The amount of these “major deviations” was 8% (IQR 0%-15%).

With regard to the outcome parameters, both, the usability and the sense of safety, showed sufficient acceptance among subjects. Of participants who were supported by either the triage algorithm or a telemedical contact, 73% (8/11) stated good or very good usability of the Smart Glasses. In addition, most subjects confirmed compatibility with the personal protective equipment in the event of a disaster. In the questionnaire’s free text, several subjects added that they felt markedly safer during the triage owing to the technical support. Only one test person regarded the assistance by a tele-senior EMS physician during the triage as superfluous, as “one does not need a doctor for triage.” This subject felt the doctor’s contact was intrusive, and he would prefer to do triage alone in the future.

**Table 2:** The demographic profile.

Group		Control group (n=20)	Triage with PRIOR^a^ service display (n=7)	Triage with tele-senior emergency medical service physician (n=4)
**Gender, n (%)**				
	Females	4 (20)	1(14)	0 (0)
	Males	16 (80)	6 (86)	4 (100)
Age (years), mean (range)		32.5 (21-50)	34.1 (28-40)	26.8 (24-31)

^a^PRIOR: Primary Ranking for Initial Orientation in Rescue.

## Discussion

### Principal Findings

In a simulated MCI scenario, Smart Glasses apps for displaying a triage algorithm or telemedical contact with an experienced emergency physician were applied to support the triage conducted by paramedics compared with a conventional control group. While in one test group, the PRIOR triage algorithm was used to ensure standardized decision support, a telemedical connection to a senior EMS physician, who could also follow a triage algorithm or could deviate from it, was provided in the second test group. Although triage took almost 2 times as long in each test group when compared with the control group, the overall quality of triage—as measured by its accuracy—was markedly increased.

The duration of a conventional triage with 16.6 seconds is consistent with similar studies. It was noticeable that many of the participants did not apply the previously learned PRIOR, but often decided spontaneously; this was also reflected in the overall bad triage quality with a huge interindividual range. Only 58% of the triage corresponded to the correct results previously determined by 3 independent, experienced emergency physicians. Thus, the accuracy of triage remains under the experience of other studies that also investigated the use of different triage algorithms [16,17].

In the observation of subjects, many displayed distinct nervousness, which, in turn, confirmed the realism of the simulation. Many went into the so-called “tunnel vision,” and the triages were wrong. For the most part, a higher priority category was chosen. While this phenomenon is known as a potential source of error for the PRIOR algorithm, this extent indicates that the algorithm has rarely been correctly applied. Although the control group was explicitly instructed to use the algorithm, none of the subjects used the handed-out pocket card, which is also available for real disaster use in many EMS units. Numerous studies showed that checklists in medicine are not well accepted but regularly used in other safety-related professions, for example, in aviation [18,19].

Finally, those subjects who viewed the PRIOR triage algorithm on the Smart Glasses app showed markedly better triage quality, which was significantly increased with 92% matches compared with 58% for the control group. All participants in this study group were introduced to use the app for algorithm display for every triage. By working through the decision tree, the subjects were forced to use the PRIOR until the triage result was achieved. Owing to the direct display of the algorithm in front of the eyes compared with the pocket card, this tool was really used. However, this resulted in a longer duration of triage, which almost doubled compared with the control group.

In this context, it is important to note that this group has digitally recorded the triage results compared with a manual tally maintained by the control group; this resulted in many advantages with decisive importance in the deployment tactics. In addition, it allowed the digital results to be retrieved from anywhere and could, in future, contribute to the early knowledge of human and material needs; this is particularly important in a disaster scenario to better plan for rare resources such as hospital capacity and ambulances. However, an internet connection is not required to display the PRIOR algorithm in Smart Glasses but required for the digital acquisition of the triage results.

The telemedical assistance in triage was generated by a live video streaming through the camera of the Smart Glasses; this showed a particularly good triage quality of 90% and thus, a highly significant increase compared with the control group. This was certainly owing to both, the four eyes principle and the presence of a doctor in the screening process. However, this contradicts the statement of the paramedic in the questionnaire who considered a doctor in the triage pointless. The much better quality of triage is certainly also attributed to the high level of compliance with the guidelines for the processing of the screening algorithm, as well as other procedural instructions; this phenomenon is also indicated by other studies of individual medical treatments by the tele-EMS physician in Aachen Germany [20].

The duration of the tele-senior EMS physician-assisted triage was markedly prolonged, but a digital acquisition of the triage results was achieved, unlike the control group. In addition, the tele-physician was able to collect and document more information in addition to the triage category, such as the patient name and first diagnoses. The longer time required for classification into the category III of the slightly injured is a well-known phenomenon in the PRIOR algorithm, as well as a high rate of overtriage [2]. The time lag from technical support was the lowest in triage category I (Figure 4). As this category identifies severely injured patients with immediate treatment priority, this is an important finding.

The reason why no 100% accuracy in triage has been achieved, however, can best be explained by the different estimation of qualitative characteristics. Thus, it must be assessed whether there is a respiratory or circulatory disorder, without this being described in more detail. In addition, the group triage algorithm display on the data glasses did not achieve full accuracy. In addition to the above reasons, errors in the menu navigation of the data glasses and thus in the course of the algorithm can have an influence here.

In 2015, a feasibility study was carried out on the app of modern telemedicine in a disaster to triage, in which only 2 patients were triaged with telemedicine [21]; the authors found no marked differences in the quality of the triage, but in the duration. In another study, the use of optical head-mounted displays in disaster missions was mentioned as beneficial, without direct comparisons to a control group [22]. Another triage algorithm was tested on Google Glass during a full-scale exercise to perform visually guided augmented-reality Simple Triage and Rapid Treatment triage and identify casualties and collect georeferenced notes, photos, and videos to be incorporated into the debriefing [23]. However, this study demonstrated for the first time the controlled randomized comparison between conventional triage, the display of triage algorithms as augmented reality, and telemedically assisted triage.

The increased quality of triage by using the Smart Glasses was also reflected in the questioning of subjects. In this study, the majority of subjects who underwent triage with the technical support provided by the Smart Glasses described an increased sense of security; this can be an important factor for the emergency services in the stressful and unfamiliar situation of a disaster case.

Sufficient usability of the Smart Glasses was confirmed under realistic conditions. Operations using the optical touchpad for menu control of the Smart Glasses was also done while wearing protective gloves, which this led to no considerable problems, although only a very short briefing was given when handling the Smart Glasses. In addition, the simulation was completed by subjects using their own personal protective equipment, and sufficient compatibility was confirmed in the questionnaire.

### Limitations

The different size of the study groups resulted from the fact that the control group was also part of a parallel observational study. The size of the 2 study groups with technical support from the data glasses (with both the display of the algorithm and telemedical contact) was originally designed with n=10. However, owing to the given timeslots, not all paramedics could participate; this fact is a limitation that should be considered in future studies.

Another limitation, in addition to the low battery capacity, which could be extended by connecting a battery pack (Figure 2), there is a lack of compatibility with personal glasses. For eyeglass wearers to be able to use the Smart Glasses, personal glasses would have to be adapted; this would involve considerable costs. In noneyeglass wearers, however, the current Smart Glasses also serve as protective eyewear. In addition, the local Wi-Fi connection led to problems. In 10 triages, no adequate connection could be achieved owing to structural obstacles; these were excluded from the study results. A mobile connection with sufficient network coverage and increased battery capacity would certainly make sense here. Unfortunately, such solutions are currently not available on the market.

### Conclusions

In summary, technically assisted triage shows markedly better quality than traditional methods. Smart Glasses have proven to be a useful tool in disaster medicine; they allow EMS responders to continue working with both hands while information is displayed on the monitor and data are collected through the integrated camera. The delay of the triage seems acceptable and in view of the digital coverage of the triage quality. Further developments to the system, as well as use in routine operations, will certainly shorten the duration of triage markedly. Assuming that both augmented and virtual reality will gain in importance in the coming years in both work and leisure, future users will then use it much more quickly and intuitively.

This study has shown that research on screening assistance procedures is still required to achieve sufficient quality of triage, which can be critical and contribute to the targeted and prioritized treatment and transport of patients in a disaster situation. High-tech can thus also support special challenges in the event of a disaster. These missions are rare and therefore lacking in routine with all emergency medical professionals. It is important to exploit all potentials of modern technologies in such situations and integrate and use augmented reality and telemedicine in existing civil defense structures.

## Appendix

Multimedia Appendix 1 CONSORT‐EHEALTH checklist (V 1.6.1).

## Abbreviations

AUDIME: Augmented Disaster Medicine
EMS: emergency medical service
IQR: interquartile range
MCI: mass casualty incident
PRIOR: Primary Ranking for Initial Orientation in Rescue service
